# Impact of virtual reality training on mastoidectomy performance: a prospective randomised study

**DOI:** 10.1007/s00405-023-08143-1

**Published:** 2023-07-28

**Authors:** Jesse Tapiala, Matti Iso-Mustajärvi, Tomi Timonen, Hana Vrzáková, Aarno Dietz

**Affiliations:** 1 School of Medicine, Institute of Clinical Medicine, University of Eastern Finland, Kuopio, Finland; 2 Department of Otorhinolaryngology, Kuopio University Hospital, Puijonlaaksontie 2, 70210, PL 100, 70029 Kuopio, Finland; 3 School of Computing, University of Eastern Finland, Joensuu, Finland

**Keywords:** Mastoidectomy, Surgical education, Virtual reality, Temporal bone surgery

## Abstract

**Purpose:**

The opportunities for surgical training and practice in the operating room are in decline due to limited resources, increased efficiency demands, growing complexity of the cases, and concerns for patient safety. Virtual reality (VR) offers a novel opportunity to enhance surgical training and provide complementary three-dimensional experience that has been usually available in the operating room. Since VR allows viewing and manipulation of realistic 3D models, the VR environment could enhance anatomical and topographical knowledge, in particular. In this study, we explored whether incorporating VR anatomy training improves novices’ performance during mastoidectomy over traditional methods.

**Methods:**

Thirty medical students were randomized into two groups and taught mastoidectomy in a structured manner. One group utilized a VR temporal bone model during the training while the other group used more traditional materials such as anatomy books. After the training, all participants completed a mastoidectomy on a 3D-printed temporal bone model under expert supervision. Performance during the mastoidectomy was evaluated with multiple metrics and feedback regarding the two training methods was gathered from the participants.

**Results:**

The VR training method was rated better by the participants, and they also needed less guidance during the mastoidectomy. There were no significant differences in operational time, the occurrence of injuries, self-assessment scores, and the surgical outcome between the two groups.

**Conclusion:**

Our results support the utilization of VR training in complete novices as it has higher trainee satisfaction and leads to at least as good results as the more traditional methods.

**Supplementary Information:**

The online version contains supplementary material available at 10.1007/s00405-023-08143-1.

## Introduction

Increased concern for patient safety and operating room efficiency, shorter workweeks and the ever-increasing complexity of the surgical cases have decreased the number of opportunities the residents have for hands-on training [1]. At the same time, contemporary surgical training has relied more on volume rather than on a specifically designed curriculum that ensures sufficient anatomical knowledge and technical skills required to become a surgeon [1]. To cope with these challenges new, more efficient, and safer training methods should be adopted [2]. Although traditional training in the operating room is irreplaceable, new technologies offer novel opportunities for the development of complementary training methods.

Numerous studies have suggested that utilizing virtual reality (VR) technology in surgical training might be beneficial [1, 2]. VR is a computer-generated, three-dimensional (3D) environment that can be explored from practically any point of view. This environment is interactable, and alterations can be made by the user utilizing an immersive stereoscopic 3D environment achieved with 3D goggles and motion sensors. VR technology offers many possibilities from 3D anatomy models to realistic simulators with automatic performance tracking and haptic feedback [1]. In medicine, the application of VR is already considered for preoperative planning, anatomical education, and technical skills practice [1, 3, 4].

In general, surgical training the effectiveness of VR simulators has been proved mainly in novices, with a positive effect on both laparoscopic and open procedures [1, 5]. In otologic surgery few studies have already shown that skills obtained with VR simulators carry over to dissection training [6–8].

While the simulators have been in the spotlight, there has been less interest in the other possibilities of VR technology. A recent meta-analysis of VR’s effectiveness on anatomical training, without the inclusion of a simulator, reported that VR increased the test scores when compared to traditional methods [9]. It seems that VR might be beneficial in formulating a better 3D understanding of complex anatomical structures such as temporal bone [3]. However, this effect might be diminished for more experienced surgeons [3]. A recent study with a large sample size found that in the training of middle ear anatomy, the VR-supported teaching method was comparable to traditional teaching methods. In addition, VR-supported teaching method was associated with higher self-reported knowledge competence scores and higher satisfaction [10].

**Image 1:**
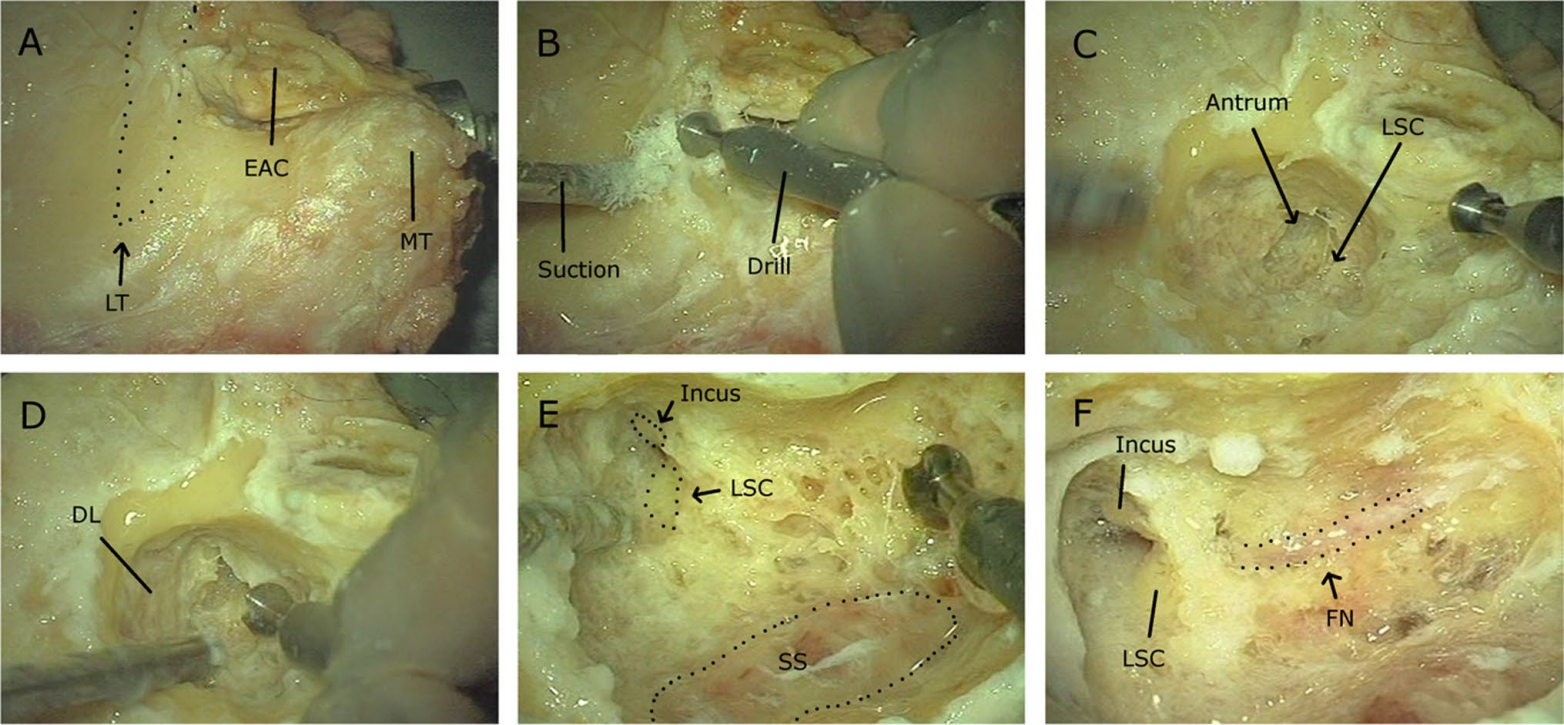
Overview of mastoidectomy on a cadaver temporal bone. **A** Bony landmarks on the surface (*LT* linea temporalis, *MT* mastoid tip and *EAC* external auditory canal). **B** Removal of the cortex and first air cells. **C** More air cells have been drilled away and the antrum and lateral semicircular canal (LSC) can be appreciated. **D** Cleaning of the dura lamel (DL). **E** Air cells have been removed over the sigmoid sinus (SS) and both incus and LSC can be seen. **F** Facial nerve (FN) has been located

Mastoidectomy (Image 1) is a otosurgical procedure where the surgeon removes the temporal bone’s air cells with a surgical drill under an operating microscope. The complex anatomy with many critical structures, small and restricted operating space, as well as the technical skills needed, make these procedures difficult. Consequently, it takes years to achieve the required knowledge and technical skills.

Presently, cadaveric temporal bone training is the gold standard for learning mastoidectomy [11]. However, cadaveric training has become more difficult and more expensive due to a restricted availability of cadaveric temporal bones [11, 12]. This has become problematic, as with any skill requiring fine motor skills, the cornerstone of training is repetition [1]. This has led to a growing interest in finding other methods, such as VR technology, to support cadaveric training. In theory, a good VR simulator and controllers with accurate haptic feedback could offer the potential for unlimited, repeatable, safe, and realistic training for mastoidectomy, posterior tympanotomy, and other surgical procedures in temporal bone. Other advantages would be better accessibility of training while significantly reducing costs as opposed to cadaver or operation room training and instant automatic feedback by the system to enhance trainee learning. Short supervised or self-directed VR training sessions have shown to increase the performance of trainees for their first traditional temporal bone dissections [6–8].

In the future, another option for hands-on training besides VR simulators could be 3D-printed anatomical models. 3D-printed temporal bones have some possible advantages to VR simulation as the haptic feedback is presumably better and genuine equipment can be used. On the other hand, VR simulation can include the soft tissues as well and repetitions do not incur extra costs. 3D-printed temporal bones have already been investigated in a few studies [13]. Despite their high ratings some concerns still exist [13]. The models are not perfect in their anatomical accuracy and the replication of authentic bone properties is still underway [13]. If perfected and validated, the 3D-printed temporal bones could potentially offer a way to replace at least some of the training currently undertaken with cadaveric bones.

In the present study, we evaluate the validity of anatomical VR training by comparing it to the traditional way of training including anatomy books, dissection guides and expert instruction in complete novices. After the training, the participants perform a mastoidectomy and their performance is evaluated. Feedback related to the training methods is also gathered. Our main hypothesis was that the VRT group would perform better based on the presumed advantage of obtaining a better 3D understanding of the anatomy. Using three objective measures (operation time, need of assistance, and the Welling scale score [14], we computed a non-weighted Z-score as the main outcome variable.

## Materials and methods

### Study design

This study was designed as a prospective randomised study. We recruited medical students with minimal experience in surgery and randomly split them into two groups: the virtual reality group (VRT) and the traditional training, control group (TT). Both groups received identical training utilizing two different methods and then performed a mastoidectomy on 3D-printed temporal bone models. Participants’ performance was evaluated after the training drilling was completed. Participants were volunteers and signed an informed consent form. There were no risks or benefits included for the participants. An organizational permit was applied for and granted by Kuopio University Hospital (permit number: 5551879).
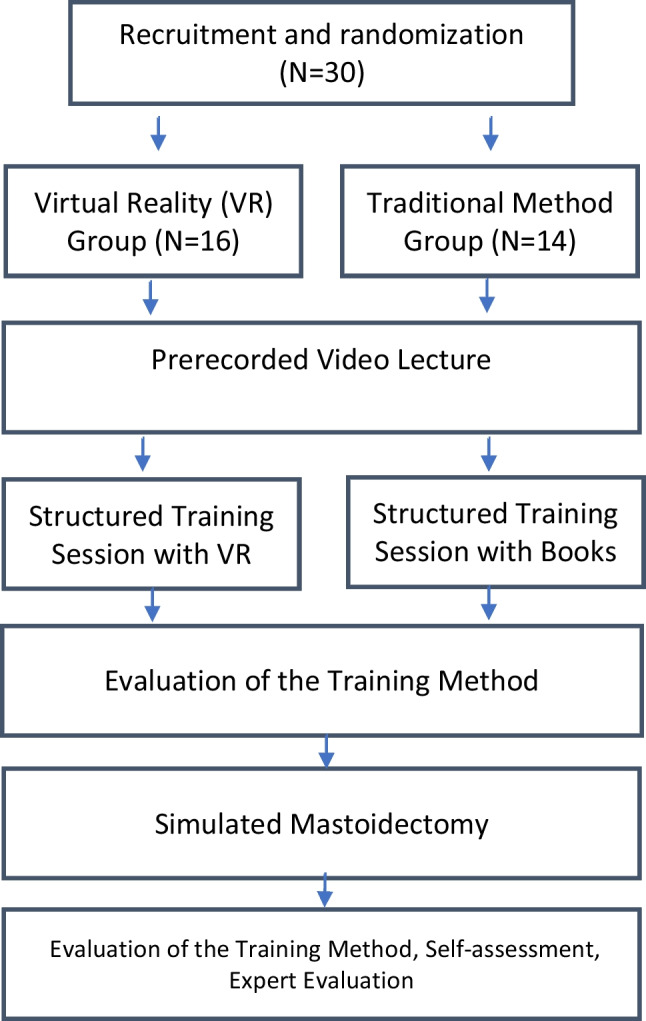


### Participants

We recruited medical students (fourth to sixth-year students) with an invitation to volunteer for our study. Medical students were selected for their uniform skillset and baseline knowledge as they were expected to have little experience with mastoidectomy and microscopic surgery. In addition, the large pool of medical students allowed for the sufficient sample size and adequate statistical power.

A total of 30 participants (16 males and 14 females) were randomly selected from volunteers and then randomly assigned into two groups with randomized identification numbers to maintain anonymity after signing an informed consent form. Table 1 shows the participants’ demographics. Most of the participants reported previous experience with VR for recreational purposes mainly.

**Table 1:** Demographics of the participants (*n* = 30) for the two groups: virtual reality group (VRT) and traditional group (TT)

		Total (*n* = 30)	VRT (*n* = 16)	TT (*n* = 14)	*p* value
Mean age		26.3 years	26.0 years	26.6 years	ns
Gender					
	Female	14	7	7	ns
	Male	16	9	7	
Class					
	4	14	6	8	ns
	5	10	7	3	
	6	6	3	3	
Dominant hand					
	Right	25	12	13	ns
	Left	3	2	1	
	Both	2	2	0	
Previous experience with surgical drilling					
	Yes	0	0	0	ns
	No	30	16	14	
Previous experience with virtual reality					
	Yes	16	9	7	ns
	No	14	7	7	
Previous experience with surgical microscope					
	Yes	24	13	11	ns
	No	6	3	3	

Mann–Whitney *U* test, Fisher’s exact test and chi-squared test were used where applicable

*Ns* not significant (*p* > 0.05)

### Mastoidectomy training

**Image 2:**
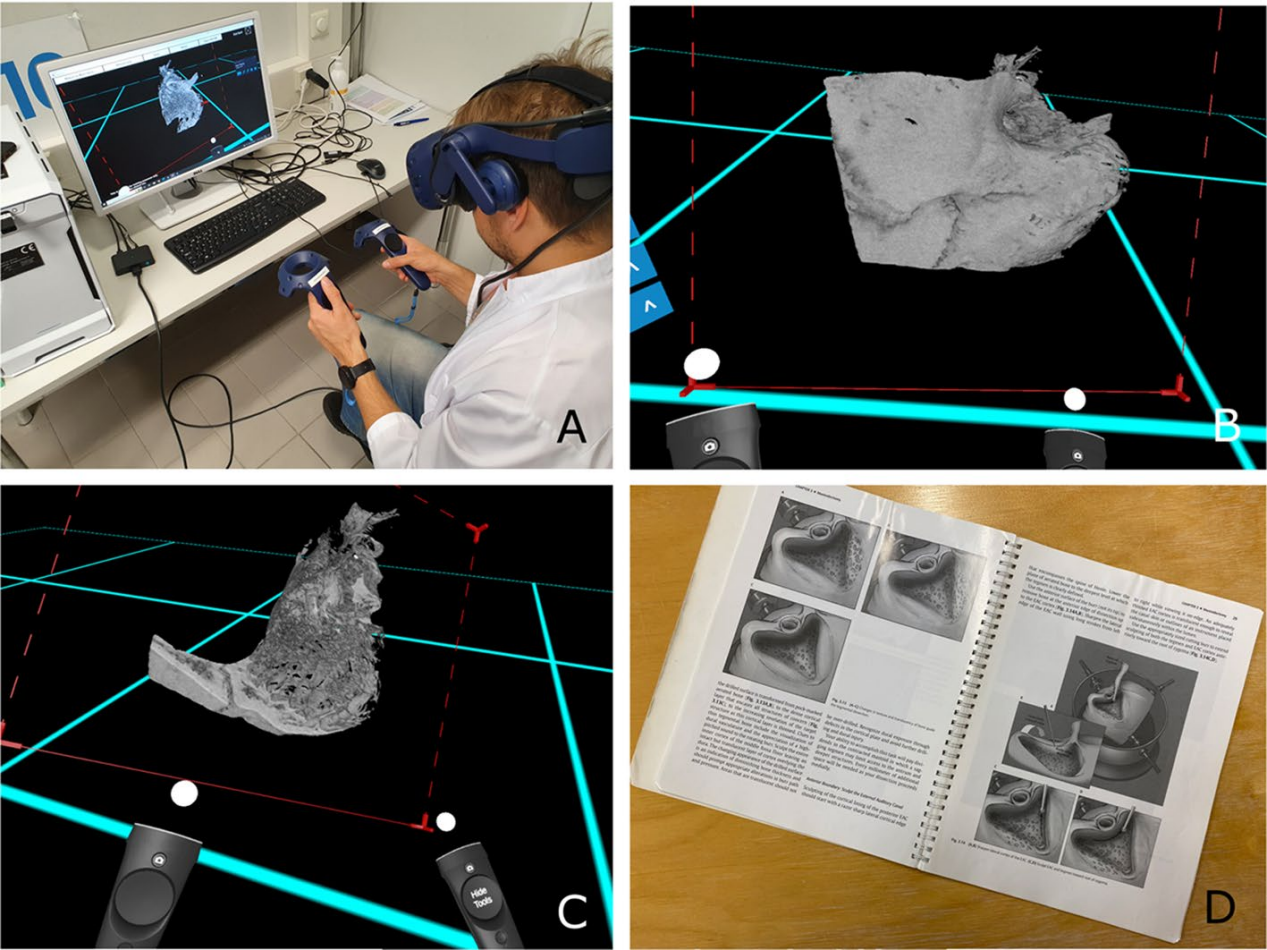
**A** The virtual reality (VR) equipment used. **B** The temporal bone model as seen in the VR environment. **C** The temporal bone can be viewed from all angles and “cut” with the cross-sectioning tool (red square). **D** Temporal bone as seen in the dissection manual used [16]

Each participant received a link to a 15 min introductory video lecture with a short overview of the relevant anatomy (Appendix 4.) and the procedure. All trainees then participated in structured training sessions in groups of three. During the 2 h long training sessions, the ENT specialist repeatedly explained the relevant anatomy and the procedure. The session included the introduction to the anatomical landmarks (Appendix 4.), their relevancy to mastoidectomy, and the steps of the procedure with respect to the subtasks defined in the next section. The trainees were instructed on how each subtask is approached in terms of general and task-specific drilling techniques and critical landmarks. The trainees could also ask any questions from the specialist. The TT group (*n* = 14) used a basic dissection guide [15] and anatomy books in their training, while the VRT group (*n* = 16) utilized a reconstructed 3D model of a temporal bone from a basic computed tomography scan in a VR environment (Image 2). The 3D temporal bone model and the VR environment were created with the Adesante SurgeryVision^™^ (Adesante Oy, Turku, Finland) software. Two controllers and a head-mounted display (HTC Vive Pro, HTC, New Taipei, Taiwan) were used to display and manipulate the model in a VR environment.

### Mastoidectomy drilling procedure

**Image 3:**
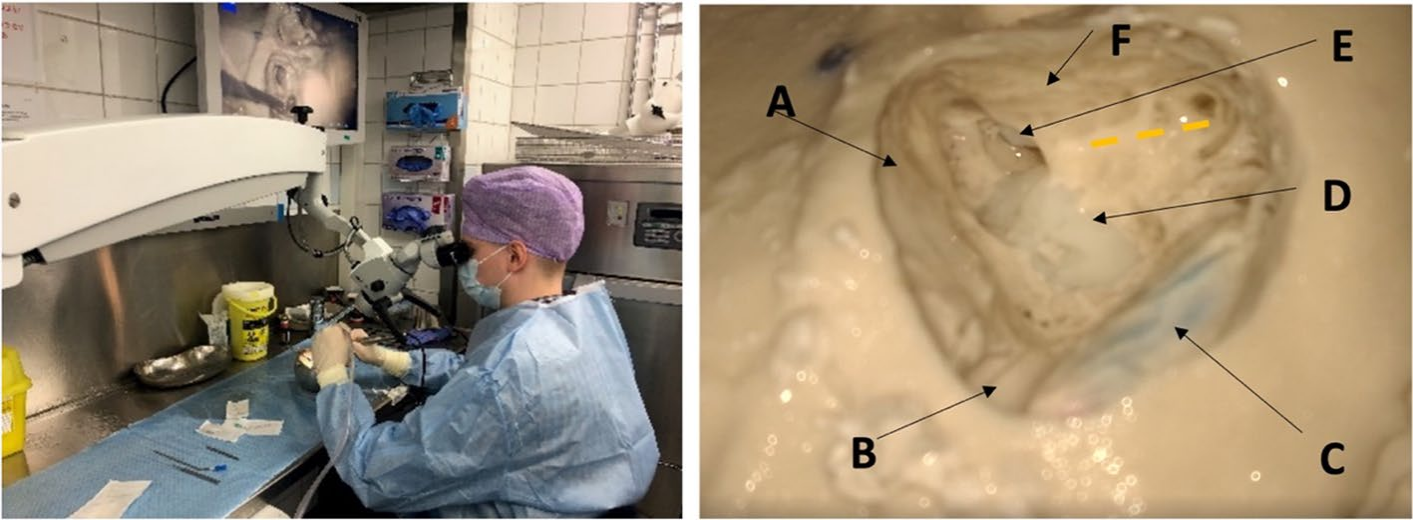
Left image: mastoidectomy drilling setup with a 3D-printed temporal bone model, a surgical microscope attached to an external screen, a surgical drill, and a suction tool. Right image: a microscopic view of a mastoidectomy in a 3D printer temporal bone. The yellow line represents the approximated location of the facial nerve under the bone. **A** = dura lamel, **B** = sinodural angle, **C** = sinus sigmoideus, **D** = lateral semicircular canal, **E** = incus, **F** = posterior ear canal wall

All participants performed a mastoidectomy on 3D-printed temporal bone models (Temporal Bone Patient “Schmidt”, Phacon, USA, Atlanta) using a surgical microscope (Zeiss Omni Pico), a surgical high-speed drill with irrigation and a suction tool (Image 3). The situation simulated an operating room environment including authentic instruments. The procedures were supervised by one of the authors. Assistance was provided only when the participant specifically asked for it. Mastoidectomy was divided coarsely into the following subtasks:

- Task 1: Identify the correct starting position from the landmarks on the surface of the temporal bone and drill away enough mastoid air cells to reveal dura lamel.
- Task 2: Identify the sigmoid sinus and the sinodural angle. Clear the air cells over the dura lamel, the sigmoid sinus and the sinodural angle.
- Task 3: Continue removal of the aircells to find the antrum.
- Task 4: Locate the lateral semicircular canal and the corpus of the incus.
- Task 5: Thin the posterior canal wall sufficiently.
- Task 6: Locate the facial nerve and finish the mastoidectomy by removing any leftover air cells.

### Factors and performance metrics

#### Evaluation of the training method

After the training session, each participant filled out a structured evaluation form (Appendix 1.) in which they evaluated the training method used. Free-form feedback was also collected about the possible advantages or the drawbacks of each method. The result of the survey was also utilized for monitoring quality control between the training sessions in each participant.

The same evaluation was repeated right after the procedure was finished.

#### Operational time and the need for assistance

During the procedure, the authors recorded the timestamps of completed subtasks related to the important landmarks using custom-made software (Python 3.9) (Appendix 4.). The software also logged the total operational time and the timestamps of participants’ requests for assistance.

#### Self-assessment

After the procedure, the participants assessed their own performance using a structured form (Appendix 2.) by Andersen et al. [16] with the exclusion of the mastoid tip and posterior tympanotomy sections. The evaluation consisted of the overall technique and outcome as well as how well they managed specific parts of the procedure.

#### Surgical outcome

A blinded evaluation of the drilled 3D-printed temporal bones was carried out by two experts using a modified Welling Scale [14]—excluding the parts not included in our training (Appendix 3). The experts also noted whether critical structures (incus, facial nerve, buttress, chorda tympani or posterior ear canal wall) were damaged. In addition, overall scores from one (worst) to five (best) were given to the temporal bones regarding the overall expression of the mastoidectomy. The scores were then compared to assess interrater reliability.

For getting an overall and the most objective evaluation of the participants’ performance, a non-weighted composite variable was formed based on *Z*-scores of the three main objective variables (time to completion, need of assistance, and the surgical outcome). In the formula, the inverse of the total Welling score was used to have positive *Z*-scores represent worse performance uniformly.

Possible correlations between the time to completion, the amount of assistance needed, the surgical outcome, the possible injuries and the self-assessments were also explored.

### Statistics

Statistical analysis was conducted in the SPSS Base 27.0 Statistical Software Package (SPSS Inc, Chicago, IL, USA). The performance of the two groups were compared with the independent samples Mann—Whitney *U* test as the sample size was quite small and normal distribution was not presumed. Two-tailed *p* values of < 0.05 were considered significant.

## Results

Overall, the participants in the VRT gave higher scores for their training method when compared with the TT control group both before and after the procedure (Table 2). In general, the scores for both methods decreased after the procedure in all the categories. However, the overall grade given for the VRT method remained the same between the evaluations and was significantly better than the grade for the TT method (*p* < 0.001) after the procedure. There were no significant differences apparent from the evaluations between the training sessions and the scores given were quite consistent between all the participants.

**Table 2:** Mean scores for the two teaching methods (virtual reality method = VRT and traditional method = TT) given by the participants

	VRT (*n* = 16)	TT (*n* = 14)	*P* value
Appearance of the anatomy (1–5 points)			
Pre	4.4 (SD 0.6)	3.9 (SD 0.6)	0.093
Post	4.3 (SD 0.8)	3.7 (SD 0.6)	0.070
Change	− 0.1 (SD 1.0)	− 0.2 (SD 0.8)	0.759
3D perception (1–5 points)			
Pre	4.3 (SD 0.8)	3.3 (SD 0.9)	0.002
Post	4.0 (SD 1.0)	2.9 (SD 1.2)	0.013
Change	− 0.3 (SD 0.7)	− 0.4 (SD 1.2)	0.697
Understanding of the anatomical structures (1–5 points)			
Pre	4.4 (SD 0.5)	3.9 (SD 0.6)	0.052
Post	4.1 (SD 0.8)	3.4 (SD 0.9)	0.070
Change	− 0.4 (SD 0.6)	− 0.5 (SD 0.9)	0.608
Understanding of the procedure (1–5 points)			
Pre	4.4 (SD 0.6)	4.0 (SD 0.4)	0.110
Post	4.1 (SD 1.0)	3.6 (SD 0.9)	0.120
Change	− 0.3 (SD 0.8)	− 0.4 (SD 0.8)	0.728
Understanding of the relationships between the anatomical structures (1–5 points)			
Pre	4.3 (SD 0.7)	3.5 (SD 0.8)	0.010
Post	4.0 (SD 1.0)	3.3 (SD 1.1)	0.085
Change	− 0.3 (SD 1.0)	− 0.2 (SD 1.0)	0.918
Total score (5–25 points)*			
Pre	21.8 (SD 1.9)	18.6 (SD 2.6)	0.001
Post	20.4 (SD 3.7)	16.9 (SD 3.3)	0.013
Change	− 1.1 (SD 2.9)	− 2.4 (SD 3.2)	0.400
Overall grade (1–5 points)^#^			
Pre	4.4 (SD 0.5)	4.0 (SD 0.6)	0.085
Post	4.4 (SD 0.6)	3.4 (SD 0.6)	< 0.001
Change	0 (SD 0.5)	− 0.6 (SD 0.5)	0.019

The evaluation was first done after the training sessions (Pre) and repeated after completing the procedure (Post). The mean change between these evaluations (change) and the categorial scores combined (Total score) were also noted. The scores of both groups were compared with the independent samples Mann–Whitney *U* test

*Sum of subcategory scores

^#^Overall 1–5 grade given for the method in general

In the open feedback, the participants described the advantages of the VRT method in terms of 3D perception and topographical anatomy. Disadvantages most often reported by the VRT group were the lack of colours and soft tissues in the model, small problems with the equipment and the view (such as a difficulty to achieve a sharp picture) as well as the difficulty of understanding the real proportions of the structures. TT method was most often thought to have easily identifiable anatomical structures and steps of the procedure. On the other hand, the 3D perception and topographical anatomy was deemed hard.

During the surgical drilling, the VRT group asked for assistance fewer times than the control group (Table 3). On average, participants in the VRT group required assistance 10.7 times (SD 5.63) while the control group did so 15.5 times (SD 7.06). The difference was statistically significant (*p* = 0.022).

**Table 3:** Mean scores for the variables used to evaluate each participants (*n* = 30) performance during the mastoidectomy in both of the groups

Variable	Virtual reality group (*n* = 16)	Traditional method group (*n* = 14)	*P* value	Total (*n* = 30)
Assistance needed (number of times)	10.7 (3.0–23.0, SD 5.6)	15.5 (7.0–31.0, SD 7.1)	0.022	12.9 (3.0–31.0, SD 6.7)
Time to completion (minutes)	75.5 (31.8–149.5, SD 31.5)	67.7 (43.8–150.2, SD 27.2)	0.498	71.9 (31.8–150.2, SD 29.3)
Total self-assessment score (6–30 points)	20.1 (13.0–29.0, SD 5.2)	19.1 (15.0–25.0, SD 2.9)	0.728	19.6 (13.0–29.0, SD 4.2)
Expert evaluation				
Overall grade from experts (1–5 points)	2.2 (1.0–4.0, SD 1.0)	2.7 (1.5–4.5, SD 0.9)	0.154	2.5 (1.0–4.5, SD 1.0)
Total Welling score (0–20 points)	10.7 (4.3–17.3, SD 3.8)	13.1 (5.5–18.9, SD 3.9)	0.142	11.8 (4.3–18.8, SD 4.0)
Injuries				
Incus injured	5 cases (31.3%)	3 cases (21.4%)	0.689*	8 cases (26.7%)
Excessive buttress removal	3 cases (18.8%)	5 cases (35.7%)	0.417*	8 cases (26.7%)
Facial nerve injury	3 cases (18.8%)	0 cases	0.228*	3 cases (10.0%)
Damage to the posterior canal wall	0 cases	1 cases (7.1%)	0.467*	1 cases (3.3%)
No injuries	8 cases (50.0%)	7 cases (50.0%)	1.00*	15 cases (50.0%)
*Z*-scores				
Assistance needed	− 0.3 (− 1.5–1.5, SD 0.8)	0.4 (-0.9–2.7, SD 1.1)	0.022	0.000 (− 1.5–2.7, SD 1.0)
Operational time	0.1 (− 1.4–2.6, SD 1.1)	− 0.1 (− 1.0–2.7, SD 0.9)	0.498	0.000 (− 1.4–2.7, SD 1.0)
Total Welling score	− 0.3 (− 1.9–1.4, SD 1.0)	0.3 (− 1.6–1.7, SD 1.0)	0.142	0.000 (− 1.9–1.7, SD 1.0)
Composite *Z*-score	0.02 (− 1.0–1.4, SD 0.7)	− 0.03 (− 0.6–1.3, SD 0.6)	0.984	0.000 (− 1.0–1.4, SD 0.6)

Minimum and maximum values as well as standard deviations (SD) are also reported inside the parentheses

*Calculated using Fisher’s exact test

**Fig. 1:**
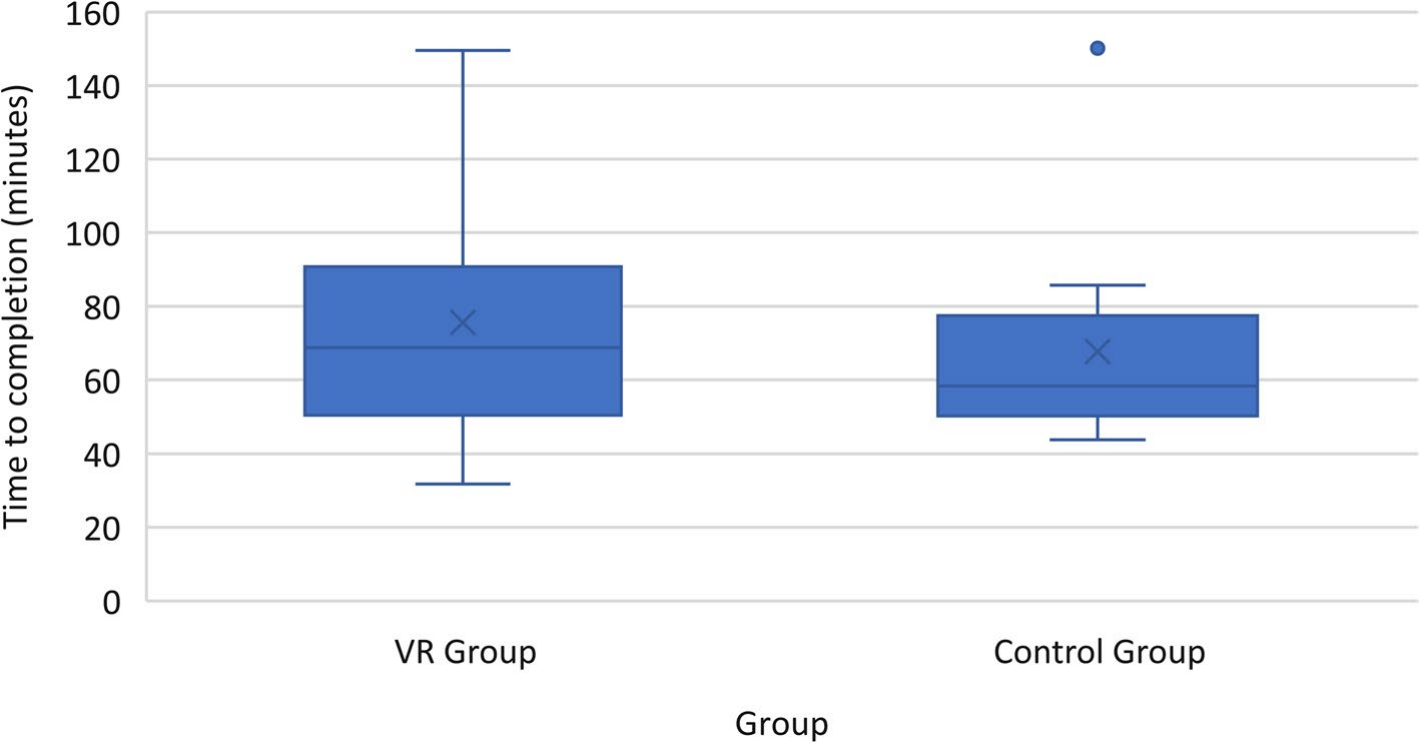
Mean time to completion for mastoidectomy in virtual reality (VR) training group and traditional method (control) training group

Every participant was able to finish the mastoidectomy under the time limit of 3 h. Overall, the VRT group took more time to complete the mastoidectomy than the control group (Fig. 1). The operational times ranged considerably and the statistical difference between groups was not significant.

The mean total score for the self-assessment scores in the VRT group was similar to the control group (Table 3).

In the expert evaluation the VRT group had lower total scores than the control group but this difference was not significant (Table 3). Similarly, the overall grade given was lower in the VRT group but again this difference was not significant. The interrater reliability was good with Pearson’s correlation coefficient being 0.739 for total scores and 0.804 for overall scores. There were significant injuries to critical structures in 50% of the cases in both groups.

The mean composite Z-score for the VRT group was 0.0227 (SD 0.680) and − 0.0259 (SD 0.624) for the TT group. This difference was not significant (*p* = 0.984) and the performance of both groups was similar (Table 3). Negative scores can be considered better as it means faster completion, the lesser need of assistance and higher scores on the Welling scale.

We also explored the possible correlations between the amount of assistance needed, operational time, self-assessment scores, expert evaluations and the occurrence of injuries (Table 4). Longer operational times seemed to associate positively with higher amounts of assistance needed. Other correlations were not noted.

**Table 4:** Possible correlations between the different performance variables were explored

Variable	Assistance needed	Time to completion	Total self-assessment score	Expert evaluation	Injuries
Assistance needed	–	*r* = 0.418 (*p* = 0.022)	*r* = − 0.201 (*p* = 0.286)	*r* = 0.025 (*p* = 0.896)	*r* = 0.112 (*p* = 0.557)
Time to completion	*r* = 0.418 (*p* = 0.022)	–	*r* = − 0.199 (*p* = 0.292)	*r* = 0.026 (*p* = 0.891)	*r* = − 0.103 (*p* = 0.589)
Total self-assessment score	*r* = − 0.201 (*p* = 0.286)	*r* = − 0.199 (*p* = 0.292)	–	*r* = 0.027 (*p* = 0.888)	*r* = − 0.097 (*p* = 0.611)
Expert evaluation	*r* = 0.025 (*p* = 0.896)	*r* = 0.026 (*p* = 0.891)	*r* = 0.027 (*p* = 0.888)	–	*r* = 0.126 (*p* = 0.507)
Injuries	*r* = 0.112 (*p* = 0.557)	*r* = − 0.103 (*p* = 0.589)	*r* = − 0.097 (*p* = 0.611)	*r* = 0.126 (*p* = 0.507)	–

Pearson’s correlation coefficient (*r*) and *p* values are reported

## Discussion

In this prospective randomized study, we taught mastoidectomy to 30 volunteer medical students with two training methods (VR and traditional) and assessed their performance using multiple subjective and objective metrics related to mastoidectomy performance. We recruited medical students without any prior experience in mastoidectomy to ensure comparable initial conditions regarding the participants’ surgical skills and knowledge. To our knowledge, this is the first prospective and randomized study to evaluate the feasibility of utilizing a 3D anatomical model in VR for training mastoidectomy without the inclusion of VR simulator training. Our goal was to explore whether VR training steepens the learning curve of mastoidectomy and if it could be utilized as a complementary method in future surgical training.

Overall, the participants rated the VR training method better than the traditional method. Consistent with previous research, the differences were most notable when considering 3D perception which was often regarded as the strength of the VR method and weakness of the TT method. Understandably, this is the clear advantage of a fully 3D model over the 2D printed images seen in the anatomy books. The individual categorical scores for both methods decreased after the mastoidectomy. This seems to indicate that in retrospect the participants felt the training did not match the real world as well as they initially thought. Despite that, the mean overall grade given for the VR method did not decrease in the second evaluation which is in clear contrast with the TT method.

Disadvantages most often reported by the VRT group were the lack of colours and soft tissues in the model, small problems with the equipment and the view (such as a difficulty to achieve a sharp picture) as well as the difficulty of understanding the real proportions of the structures. In the future it is likely that technical advancements can amend at least some of these concerns.

Even though the VRT group required less assistance during the operation, they were not faster nor was their outcome better than the TT group and they made a similar number of mistakes. We expected that a possible increase in understanding of the mastoidectomy prior to drilling would reflect in the number of assistance requests, operational time and the number of mistakes as well as the outcome. Based on our results this was not the case and it is unclear whether the difference in the amount of assistance needed reflects better knowledge obtained. The numerous injuries observed here could point to insufficient ability to identify and understand where the critical structures are or that the technical skills were not adequate for the task. Of course, there is still the possibility that one of the methods gave a superior understanding of the anatomy and the procedure, but the participants did not have the technical skills to match that.

To holistically understand each participants’ performance, a non-weighted composite variable was created based on the operational time, the amount of assistance needed and the Welling score. These variables were chosen as a simplified indication of a successful surgery as an experienced surgeon should be able to perform a routine procedure in a timely manner, without any help and with excellent results. Evaluating surgical performance based on operational time and technical adequacy is supported by current data. For example, longer operational times have been shown to increase the risk of surgical infections and other complications leading to a worse outcome for the patient [17, 18]. Similarly, structured evaluation of the surgery’s technical adequacy intraoperatively seems to also correlate with the outcomes of the operation [19]. The amount of assistance needed is not a relevant indicator for more experienced surgeons during real-life surgeries but can still be useful in the context of novice training. We believe that by utilizing a composite variable it is possible to evaluate participants’ performances more accurately during the training than by just evaluating the different metrics individually since the surgery is highly dynamic in nature and no single variable can represent the rich and complex aspects of surgeon’s performance without taking other variables into account at the same time. With the composite variable and the *Z*-scores, it is quite feasible to put each participants’ performance into perspective when no clear benchmarks for each variable exist for our study’s population.

Our findings show that the VR method did not seem to significantly overcome the traditional training methods. At the same time, there were no notable advantages for the traditional methods either. It is also clear that the participants preferred the VR method over the traditional books and this aspect should be accounted for in the future development of mastoidectomy training. These results are in line with previous research [20, 21] although some data seems to suggest that the VR method leads to better results [3].

While VR is getting more adopted in anatomy training, evaluation of its outcomes and effectiveness in previous studies have been limited to written exams instead of a simulated procedure [20]. Therefore, a direct in-depth comparison to our findings is unfeasible with this sample. According to a translational outcome classification by Patel et al., our study corresponds to a level 2 outcome study [2]. To our knowledge, in the otologic field no study has reliably been able to show the impact of VR training (with simulator training or without) in the operating theatre or beyond (i.e., level of effectiveness > 2 as per McGaghie’s translational outcome classification) [2]. One small study (*n* = 10) explored real-life performance during an antro-mastoidectomy after VR simulator training [22]. However, only half of the participants were able to finish the supervised surgery and it is unclear how much guidance the participants had during the operation. Interestingly, multiple studies have already achieved this checkpoint in the neurosurgical field [2, 23, 24].

The clear strength of our study is the randomized and prospective approach together with the inclusion of the many different metrics of performance and methods proposed in the literature and combining them all together in this single study for the first time. While our sample size remains quite small in absolute terms, it is one of the largest when compared to earlier studies conducted on similar subjects. Our study is also the first one to explore the usage of VR for purely anatomical and theoretical training of mastoidectomy without the need for a more costly VR simulator. A recent meta-analysis on VR simulators in temporal bone surgery shows promising results for simulator training [25].

Further research will focus on study replication in a typical training situation with the repeated training sessions and increased sample size of residents. In this study, the surgical training was conducted after only a single instruction session. Repeated instruction and surgical sessions might reveal changes in participant’s learning curve over time.

In addition, recruiting residents instead of medical students will open new, relevant insights into the use of VR in their training. For example, residents’s initial anatomical knowledge and relevant surgical skills would likely vary within the group and differ greatly compared to the current sample size of medical students.

Due to the low number of ENT residents in Finland, it was unfeasible to recruit a sufficient population of residents in this study. Nevertheless, participants with minimal surgical background allowed us to examine the hypothesized benefits of VR, especially in terms of obtaining improved topographical understanding and increased performance in their first mastoidectomy. Although mastoidectomy is undeniably a complex and demanding procedure for a senior resident and even more so for medical students, the selected procedure and training setup provided the recruited participants with additional advantages.

Mastoidectomy drilling with 3D-printed bones represented a genuine surgical procedure that provided an authentic yet safe practice ground that could be easily replicated for all participants. In addition, the surgical training was completed in the training center that strongly resembled operating room setting. Taken together, medical students could experience complex surgical training in close-to-authentic conditions and ahead of their potential residency.

With respect to the research questions, the complex procedure allowed us to examine impact of VR in the highly authentic tasks, as opposed to traditional, simplified training. The complex training also contributed to research on skill transfer (i.e., how simulation training transfers to authentic operations), which is understudied in the current research on surgical innovations and training. In future work, we will also systematically evaluate participant’s surgical performance with respect to the selected model (i.e., cadaveric and 3D-printed models) and factor in participants’ personal traits and technical skills to examine the impact of training method.

Originally, we had planned to also analyse the operational times of the individual steps of the procedure. Due to the dynamic nature of mastoidectomy, these steps were completed in order that varied across participants. To provide the most authentic experience, we did not enforce a particular order of the individual steps such that other performance variables were not compromised. Due to the variable order and the sample size, the analysis of the individual steps would be misleading.

In conclusion, the VR method seems to be at least as good as the traditional method when considering the training of a complex and demanding surgical procedure for novices with the added benefit of increased trainee satisfaction.

### Supplementary Information

Below is the link to the electronic supplementary material.

Supplementary file1 (DOCX 104 KB)

## Data Availability

The datasets used and/or analysed during the current study are available from the corresponding author on reasonable request.
