# Supplementary material for: Impact of virtual reality training on mastoidectomy performance: a prospective randomised study
Source: Eur Arch Otorhinolaryngol. 2023 Jul 28;281(2):701–10. doi: 10.1007/s00405-023-08143-1 (PMC10796652; doi:10.1007/s00405-023-08143-1)
Supplement: Supplementary file 1 — Supplementary file1 (DOCX 104 KB) [file 405_2023_8143_MOESM1_ESM.docx]

Appendix 1.

**Post-training survey Hours: __________________________**

**ID-number: ______________________ Date: ___________________________**

**Scale:**

1. **Completely disagree / not realistic / not useful**
2. **Somewhat disagree / somewhat not realistic /somewhat not useful**
3. **Neutral**
4. **Somewhat agree / somewhat realistic /somewhat useful**
5. **Completely agree / realistic / useful**

**VR-system:**

1. **Appearance of anatomical structures points: _______________________**
2. **Depth perception points: _______________________**
3. **Understanding of anatomical structures points: _______________________**
4. **Learning of the procedure points: _______________________**
5. **Understanding the relationships of anatomical points: _______________________ structures**
6. **Overall grade points: _______________________**

**Advantages of VR-system:**

**__________________________________________________________________________________________________________________________________________________________________________________________________________________________________________________________________________________________________________________________________________________________________________________________________________________________**

**Disadvantages of VR-system:**

**__________________________________________________________________________________________________________________________________________________________________________________________________________________________________________________________________________________________________________________________________________________________________________________________________________________________**

**Other feedback: __________________________________________________________________________________________________________________________________________________________________________________________________________________________________________________________________________________________________________________________________________________________________________________________________________________________**

Appendix 2.

**Self-assessment form**

**ID-number: ______________________ Date: ___________________________**


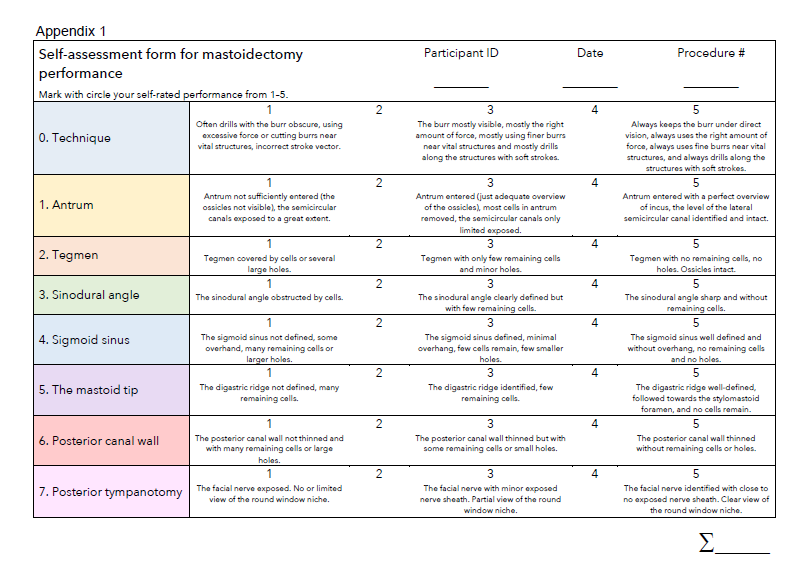


Andersen SAW, Guldager M, Mikkelsen PT, Sørensen MS. The effect of structured self-assessment in virtual reality simulation training of mastoidectomy. European Archives of Oto-Rhino-Laryngology [Internet]. 2019;276(12):3345–52. Available from: https://doi.org/10.1007/s00405-019-05648-6

Appendix 3.

**Welling Scale for Temporal Bone Dissection (WS1)**

| Please grade each item. 0 = incomplete, inadequate dissection, 1 = complete, adequate | | | | | |
| --- | --- | --- | --- | --- | --- |
| **Cortex** | | | | | |
| 1. | Cortex rounded at linea temporalis | **0** | **1** | | |
| 2. | Cortex rounded from linea temporalis to middle cranial fossa | **0** | **1** | | |
| 3. | Thinning of posterior canal wall | **0** | **1** | | |
| 4. | Complete saucerization | **0** | **1** | | |
| **Tegmen mastoideum** | | | | | |
| 5. | Dissection parallels curve of the dura | **0** | **1** | |  |
| 6. | Completely exposed | **0** | **1** | |  |
| 7. | No holes | **0** | **1** | |  |
| 8. | No cells remain | **0** | **1** | |  |
| **Sigmoid Sinus** | | | | | |
| 9. | No holes | **0** | **1** | |  |
| 10. | No cells | **0** | **1** | |  |
| 11. | No overhang | **0** | **1** | |  |
| **Sinodural Angle** | | | | | |
| 12. | Sharp | **0** | **1** | |  |
| 13. | No cells remaining | **0** | **1** | |  |
|  | | | | | |
|  |  |  |  | |  |
|  |  |  |  | |  |
| **External Auditory Canal** | | | | | |
| 16. | Canal Wall Up | **0** | **1** | |  |
| 17. | Without holes | **0** | **1** | |  |
| 18. | Without cells | **0** | **1** | |  |
|  | | | | | |
|  |  |  |  | |  |
|  |  |  |  | |  |
|  |  |  |  | |  |
|  |  |  |  | |  |
| **Facial Nerve** | | | | | |
| 23. | Identification of nerve | **0** | **1** | |  |
|  |  |  |  | |  |
|  |  |  |  | |  |
|  |  |  |  | |  |
| 27. | No exposed nerve sheath | **0** | **1** | |  |
| 28. | Identification of chorda tympani or stump | **0** | **1** | |  |
| 29. | Facial Recess Completely exposed | **0** | **1** | |  |
|  | | | | | |
|  |  |  |  |  |  |
|  |  |  |  |  |  |
|  |  |  |  |  |  |
|  |  |  |  |  |  |
|  |  |  |  |  |  |
|  |  |  |  |  |  |
|  |  |  | |  |  |
|  | | | | | |

OVERALL SCORE (1-5):

Adapted from: Butler NN, Wiet GJ. Reliability of the Welling scale (WS1) for rating temporal bone dissection  performance. Laryngoscope. 2007 Oct;117(10):1803–8.

Appendix 4. Anatomical structures of the temporal bone included in the training for both groups.

| **Surface landmarks** |
| --- |
| External ear canal |
| Spine of Henle |
| Linea temporalis |
| Zygomatic root |
| Mastoid tip |
| **Landmarks during the mastoidectomy** |
| Antrum |
| Dura lamel |
| Sigmoid sinus |
| Sinodural angle |
| Corpus of incus |
| Facial nerve |
| Chorda tympani |
| Lateral semicircular canal |
| **Other structures included in the training** |
| Superior semicircular canal |
| Posterior semicircular canal |
| Vestibulum |
| Malleus |
| Stapes |
| **Relationships between the structures** |
| Distance from sinus sigmoideus to posterior canal wall |
| Distance from the surface to the antrum |
| Distance from the surface to the incus |
| Distance from the surface to the facial nerve |
